# Modeling the COVID-19 epidemic in Croatia: a comparison of three analytic approaches

**DOI:** 10.3325/cmj.2022.63.295

**Published:** 2022-06

**Authors:** Ante Lojić Kapetanović, Marina Lukezić, Ajka Pribisalić, Dragan Poljak, Ozren Polašek

**Affiliations:** 1 Faculty of Electrical Engineering, Mechanical Engineering, and Naval Architecture, Split, Croatia; 2 Department of Public Health, University of Split School of Medicine, Split, Croatia; 3 Algebra University College, Zagreb, Croatia

## Abstract

**Aim:**

To facilitate the development of a COVID-19 predictive model in Croatia by analyzing three different methodological approaches.

**Method:**

We used the historical data to explore the fit of the extended SEIRD compartmental model, the Heidler function, an exponential approximation in analyzing electromagnetic phenomena related to lightning strikes, and the Holt-Winters smoothing (HWS) for short-term epidemic predictions. We also compared various methods for the estimation of R0.

**Results:**

The R0 estimates for Croatia varied from 2.09 (95% CI 1.77-2.40) obtained by using an empirical post-hoc method to 2.28 (95% CI 2.27-2.28) when we assumed an exponential outbreak at the very beginning of the COVID-19 epidemic in Croatia. Although the SEIRD model provided a good fit for the early epidemic stages, it was outperformed by the Heidler function fit. HWS achieved accurate short-term predictions and depended the least on model entry parameters. Neither model performed well across the entire observed period, which was characterized by multiple wave-form events, influenced by the re-opening for the tourist season during the summer, mandatory masks use in closed spaces, and numerous measures introduced in retail stores and public places. However, an extension of the Heidler function achieved the best overall fit.

**Conclusions:**

Predicting future epidemic events remains difficult because modeling relies on the accuracy of the information on population structure and micro-environmental exposures, constant changes of the input parameters, varying societal adherence to anti-epidemic measures, and changes in the biological interactions of the virus and hosts.

Epidemiological modeling is one of the main tools in infectious disease epidemic management. The recent COVID-19 pandemic is no exception, despite the prevalent neglect of evidence-based medicine principles (1,2). One of the most commonly used tools for epidemiological modeling are compartmental models, which assume that the dynamics of an epidemic depends on several discrete states, including susceptible, infected, and recovered status (3). The main advantage of these models includes the possibility of predicting the overall epidemic pattern, and their main limitation is heavy dependence on the input parameters (4,5). Numerous other approaches have been used for this purpose, relying on exploring historical patterns in predicting future events (6). The main disadvantage of such models is dependence on the initial parameters and the inability to capture the timely and relevant information in the population, which raises the need for reliable predictive tools that depend less on the starting assumptions.

Therefore, we aimed to compare various methodological approaches to epidemic prediction, with a particular focus on the performance of methods that do not require numerous inputs and rely less on the initial assumptions.

## Material and methods

We used the national COVID-19 data obtained from the ECDC (*https://www.ecdc.europa.eu/)* as the primary data source, with the analyzed period spanning 26 months, from February 2020 to April 2022, covering several well-documented complete COVID-19 outbreaks.

Three analytic approaches were used: the compartmental model (SEIRD), the Heidler function, and the Holt-Winters model (HWS). The SEIRD model development was based on three main assumptions: a stable overall population without major demographic events, population homogeneity, and that the exposed individual is infectious during the whole incubation period (Supplementary Material(Supplementary Material)).

Heidler function is an exponential approximation used to analyze electromagnetic phenomena related to lightning strikes (7). Although it is an interesting tool for electromagnetics, it was not previously used in epidemic disease modeling. There were no underlying assumptions for using the Heidler model since it relies solely on the input data.

Holt-Winters smoothing (HWS) is a decomposition method that splits the time series data into a narrow-sense trend, seasonal component, and residual (8-10). The main advantages of this approach are low input requirements (since the method solely relies on the time series data) and the ability to offset weekly or in similar regular cycle variation. Despite a known disadvantage of lags in predicted peaks due to smoothing, very precise short-term trend predictions are often reported (11-13) (Supplementary Material(Supplementary Material )).

The goodness-of-fit analysis (GoF) was based on the S value, defined as the standard error of the regression (occasionally also reported as the standard error of the estimate), which is preferred for nonlinear systems. The GoF for HWS was based on mean absolute percent error (MAPE). The SEIRD and the Heidler model were developed by using an open-source CoroPy Python package (Supplementary Material(Supplementary Material)), while HWS estimates were calculated in R.

## Results

Two schemes for the SEIRD model fitting were used – the first considered the entirety of the available data (the total number of infectious, recovered, and deceased individuals over time), and the second considered only the total number of infectious individuals over time. The first SEIRD model provided reasonably good GoF (S = 153.17; Supplementary Figure 1(Supplementary Figure 1)); the MCMC analysis indicated that subtle changes in sensitivity and specificity of the virus testing process could substantially affect the final model result. The second SEIRD model achieved a better overall fit (S = 73.08; Supplementary Figure 2(Supplementary Figure 2)), but it lacked generalizability since it relied only on a fraction of the data explaining the epidemic wave. Additionally, multi-wave SEIRD fitting was also performed regarding the total number of infectious, recovered, and deceased individuals over time. Rough transitions of the dynamics of the disease, particularly between individual epidemic waves, were captured with the overall unsatisfactory fit (Supplementary Figure 3(Supplementary Figure 2)).

The lower bound of the R0 value for the first seven months of epidemic obtained from the expected SEIRD model was 2.09 (95% CI 1.77-2.40). The upper bound R0 value was 2.28 (95% CI 2.27-2.28), determined by assuming an exponential outbreak at the very beginning of the SARS-CoV-2 epidemic and fitting the curve of the increase in the number of newly infected to a simple time-dependent analytical expression derived from the SEIRD model: , where  is the transmission rate and  is the infectivity period in days.

The use of the Heidler function yielded an exceptional fit with the first epidemic wave (S = 23.32; Supplementary Figure 4(Supplementary Figure 4)). An extension of the model for the recurrent lightning strikes had the best fit for multiple waves of the epidemic (S = 44.54; Figure 1).

The HWS was based on triple smoothing and additive model, with seven days of cyclicity. Two approaches were used, one with the daily number of predicted new cases and the other with the cumulative number of cases, both with 14 days of prediction period (starting with February 24), with daily predictions calculated for the period until April 30 (Supplementary Material(Supplementary Material)). The cumulative approach had a substantial supremacy, with MAPE of 2.7% as opposed to as much as 46.8% in the case of the daily number of new cases (Supplementary Material(Supplementary Material)). Notably, the forecast depended strongly on the number of daily cases, as MAPE started to rise with the declining number of new daily cases (Supplementary Material(Supplementary Material)).

## Discussion

Each predictive model had distinct advantages and limitations and each may contribute specific knowledge. Neither model performed well across the entire observed period, which was characterized by multiple wave-form events, influenced by the re-opening for the tourist season during the summer, mandatory masks use in closed spaces, and numerous measures introduced in retail stores and public places. Therefore, the most salient message of this study is that any single predictive model is subpar to the multi-model approach. Although this may seem like the results dilution, an in-depth understanding of the advantages and limitations of such models may bring advantages for forecasting, but also for now-casting (14). This can be especially useful in short-term predictions, where adherence to a specific model may show the true nature of the current epidemic pattern and facilitate the decision-making process (15).

Several fundamental problems burden more in-depth modeling of the epidemic risk in Croatia. The lack of reliable primary demographic and population-level mobility data are one of the most fundamental problems, requiring systematic data collection and adjustment. Furthermore, no monitoring system could estimate the current adherence to the protective measures in real-time. Interestingly, one study showed that behavioral factors and risk perception play a role in individual risk estimation (16), suggesting that epidemiological history remains an essential tool in the epidemic monitoring and control. The overall epidemic data may strongly depend on the testing approach and capacity (17), which can generate further modeling difficulties. The use of routinely collected data without harmonization is another source of problems since different institutions may have incomparable disease management practices, requiring national data and process harmonization before more definitive comparative analyses can be made.

Overall, the results of this study suggest that successful predictive epidemic modeling requires numerous sources of data, constant validity assessment, and regular updates to deliver the relevant data that can be used to steer the anti-epidemic measures.
