# Supplementary material for: Modeling the COVID-19 epidemic in Croatia: a comparison of three analytic approaches
Source: Croat Med J. 2022 Jun;63(3):295–8. doi: 10.3325/cmj.2022.63.295 (PMC9284011; doi:10.3325/cmj.2022.63.295)

Supplementary Figure 2. MCMC sampling of SEIRD-based simulation performed for the initial epidemic wave using only the number of infected cases (loss function as defined in (A12)).

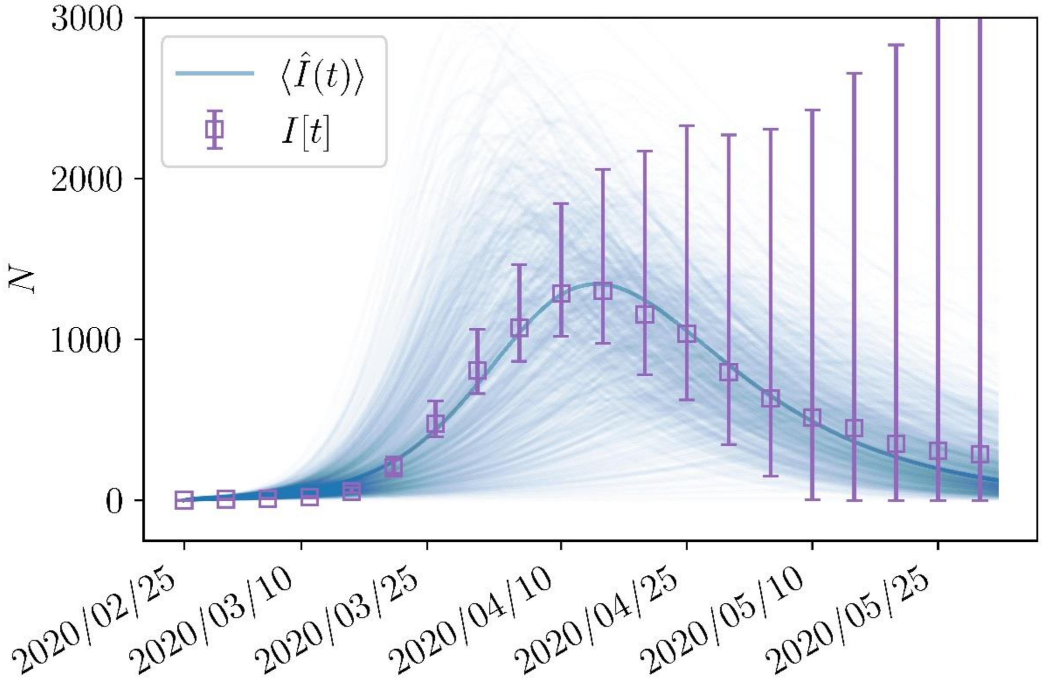

Supplement: Supplementary Figure 2 [file CroatMedJ_63_s016.pdf]
