# Supplementary figures and images for: Modeling the COVID-19 epidemic in Croatia: a comparison of three analytic approaches
Source: Croat Med J. 2022 Jun;63(3):295–8. doi: 10.3325/cmj.2022.63.295 (PMC9284011; doi:10.3325/cmj.2022.63.295)

**Supplementary Figure 3.** A multi-wave SEIRD mode.

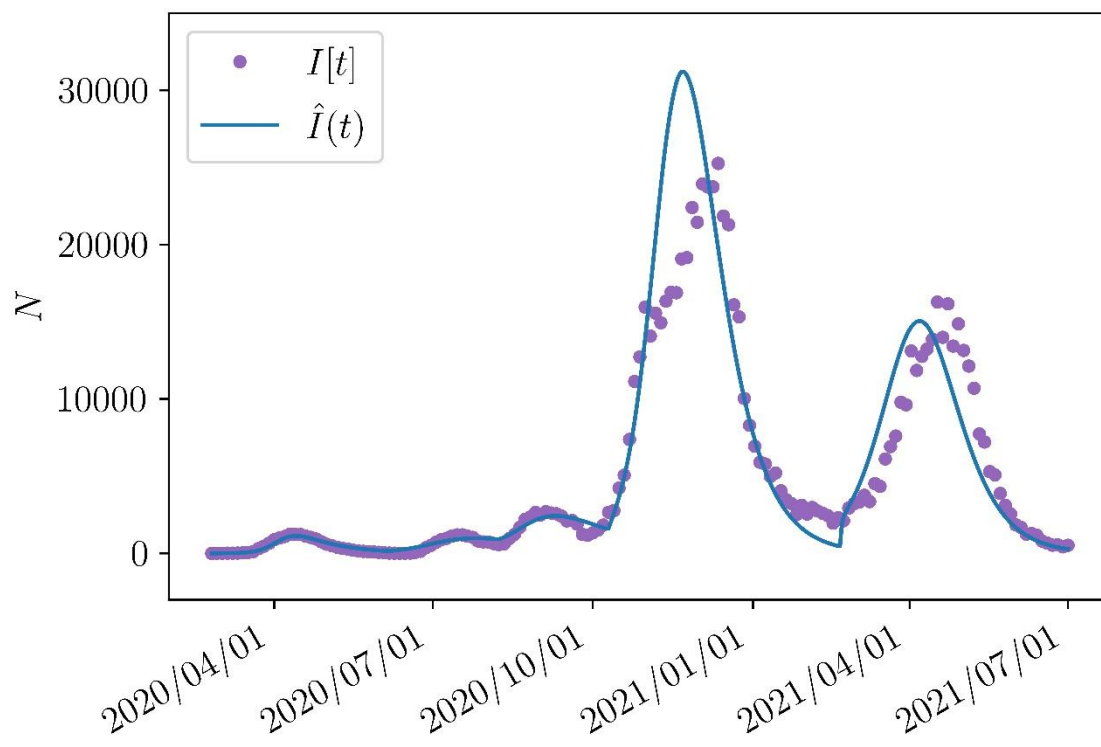

Supplement: Supplementary Figure 3 [file CroatMedJ_63_s017.pdf]

**Supplementary Figure 4.** Heidler exponential fitting of the initial epidemic wave.

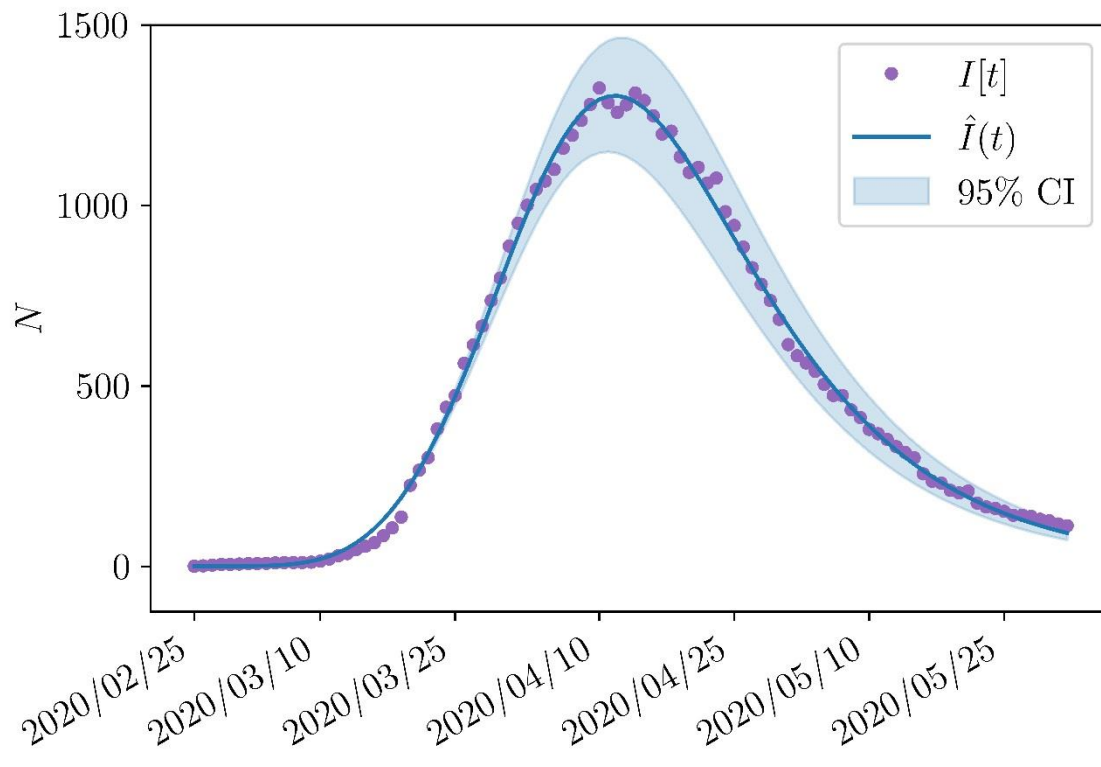

Supplement: Supplementary Figure 4 [file CroatMedJ_63_s018.pdf]
