# Supplementary material for: Modeling the COVID-19 epidemic in Croatia: a comparison of three analytic approaches
Source: Croat Med J. 2022 Jun;63(3):295–8. doi: 10.3325/cmj.2022.63.295 (PMC9284011; doi:10.3325/cmj.2022.63.295)
Supplement: Supplementary Material [file CroatMedJ_63_s019.pdf]

Supplementary Material

Appendix A: Fitting the SEIRD model to available data

Two models have been used throughout the study – the SEIRD model fitted using the confirmed number of infected individuals and the SEIRD model fitted using the confirmed number of active infected, estimated recovered and confirmed deceased individuals, respectively.

The variant of the SEIRD model in this study is an extension of the Susceptible-Infected-Recovered (SIR) model, introduced and mathematically derived in [10]-[12]. The SEIRD model is a compartmental, population-based epidemiological model used for an appropriate deterministic representation of the underlying dynamics of an infectious disease [13]. It is of utmost importance to emphasize the major assumption of the model- a homogeneous mixing of the infected and susceptible population. Furthermore, the population is assumed as the effective population constant in time, which means that no vital dynamics are considered.

The SEIRD model divides the overall effective population into five compartments; susceptible (S), exposed (E), infected/infectious (I), recovered (R) and deceased (D) compartment. The change of each compartment is a direct consequence of the dynamics of the disease and can be mathematically defined as a set of coupled nonlinear ordinary differential equations (ODEs) as follows:

$$\frac{dS(t)}{dt} = -\frac{\beta S(t)I(t)}{N} - \delta S(t)E(t)\#(A1)$$

$$\frac{dE(t)}{dt} = \frac{\beta S(t)I(t)}{N} - \alpha E(t) + \delta S(t)E(t)\#(A2)$$

$$\frac{dI(t)}{dt} = \alpha E(t) - \gamma I(t) - \mu I(t)\#(A3)$$

$$\frac{dR(t)}{dt} = \gamma I(T)\#(A4)$$

$$\frac{dD(t)}{dt} = \mu I(T)\#(A5)$$

where the total population at any time, *t*, is given by:

$$N = S(t) + E(t) + I(t) + R(t) + D(t)\#(A6)$$

Note that parameter β stands for the contact (transmission) rate or the probability of the disease transmission between a susceptible and an infectious individual; δ can be considered as the rate of asymptomatic pathogen carriers; α represents the incubation rate of exposed individuals becoming infected; γ is the recovery rate, which can be directly determined as the reciprocal value of the average duration of recovery, τ_d, and finally, μ represents the death rate. The additive feedback term $\delta\, S(t)\, E(t)$ in (1), introduced in [14], allows exposed individuals to shed the pathogen onto susceptible individuals, which is, as a matter of fact, one of the main clinical features of COVID-19.

Due to the discrete-time nature of the problem, where the period of two adjacent data points is considered as one day, ODEs (A1) to (A5) are transformed into a set of difference equations discrete in time, given as follows:

$$\frac{\Delta S(t)}{\Delta t} = \frac{S(t+\Delta t) - S(t)}{\Delta t} = -\frac{\beta S(t)I(t)}{N} - \delta S(t)E(t)\#(A7)$$

$$\frac{\Delta E(t)}{\Delta t}=\frac{E(t+\Delta t)-E(t)}{\Delta t}=\frac{\beta S(t)I(t)}{N}-\alpha E(t)+\delta S(t)E(t)\#(A8)$$

$$\frac{\Delta I(t)}{\Delta t}=\frac{I(t+\Delta t)-I(t)}{\Delta t}=\alpha E(t)-\gamma I(t)-\mu I(t)\#(A9)$$

$$\frac{\Delta R(t)}{\Delta t}=\frac{R(t+\Delta t)-R(t)}{\Delta t}=\gamma I(T)\#(A10)$$

$$\frac{\Delta D(t)}{\Delta t}=\frac{D(t+\Delta t)-D(t)}{\Delta t}=\mu I(T)\#(A11)$$

It is worth noting that the previously outlined SEIRD model setup (A7) to (A11) allows the application of a numerical solver for an initial value problem to obtain a nonlinear dependence of the solution in time, as this type of dynamical system is analytically unsolvable.

Since the parameters of the SEIRD model (*β*, *δ*, *α*, *γ*, *μ*) are unknown, the inverse system identification is carried out based on the measured COVID-19 data. SEIRD models are fitted iteratively using minimizing the loss function by using an extension of the well-known limited-memory Broyden-Fletcher-Goldfarb-Shanno (L-BFGS) algorithm that handles bound constraints on free parameters.

The choice of the loss function directly depends on the model and is defined as the L_2-norm between the approximated number of infected individuals over time and the confirmed number of infected individuals:

$$J(\beta,\delta,\alpha,\gamma,\mu)=\left|I(t)-\hat{I}(t)\right|_2\#(A12)$$

If one considers the difference between the approximated number of infected individuals over time and the confirmed number of infected individuals in summation with the difference between the approximated number of recoveries and the confirmed number of recoveries and with the difference between the approximated number of deaths and the confirmed number of deaths, the final loss function is expressed as follows

$$J(\beta,\delta,\alpha,\gamma,\mu)=\left|I(t)-\hat{I}(t)\right|_2+\left|R(t)-\hat{R}(t)\right|_2+\left|D(t)-\hat{D}(t)\right|_2\#(A13)$$

where $\hat{I}(t)$, $\hat{R}(t)$ and $\hat{D}(t)$ are the approximated number of infected individuals, recovered individuals and deceased individuals, respectively. A new set of approximations is obtained using the fourth-order Runge-Kutta initial value problem solver for each iteration of the optimization procedure. This process is repeated until convergence of the loss function to its minimum until optimal values of unknown epidemic parameters in the SEIRD model are found.

The previously outlined loss functions allow the convergence for simple rise-and-fall epidemic phenomena. However, due to the complex nature of SARS-CoV-2, which is characterized by multiple outbreak wave-form events influenced by numerous factors over a long period, convergence is not achievable. Instead, multi-wave fitting is performed for the recurrent epidemic outbreaks by using the loss function as defined in (A13). The only additional prior information needed are effective dates of the disease dynamics transitions.

Additionally, uncertainty quantification of the SEIRD output is undertaken by using the Markov chain Monte Carlo (MCMC) method. The goal is to examine how the variability of fitted parameters affects the overall output of the model by treating the input parameters of the model as random variables. Again, each compartment is considered a function of time, where the sum of all compartments is

equal to the population number. Unknown epidemic input parameters are treated as uniformly distributed random variables with expected values obtained through inverse system identification using L-BFGS-B optimization of the loss function defined as the difference between *a priori* known numbers of infectious, recovered, and deceased individuals and associated predicted curves obtained via the SEIRD model. Note that the variation coefficient (standard deviation or spread) is defined arbitrarily and is fixed to 0.2 in this study.

Appendix B: Fitting the Heidler exponential function to data

Observing the wave-forms obtained by the SEIRD model, one concludes that the successful fitting could also be carried out by using power functions combined with exponential decay functions given in [16, 17]:

$$y(t) = \frac{y_{max}}{\eta} \frac{a_1\left(\frac{t}{T}\right)^{k_1} + a_2\left(\frac{t}{T}\right)^{k_2} + \cdots + \left(\frac{t}{T}\right)^{n}}{b_0 + b_1\left(\frac{t}{T}\right)^{m_1} + b_2\left(\frac{t}{T}\right)^{k_2} + \cdots + \left(\frac{t}{T}\right)^{n}} e^{-t/\tau} \#(B1)$$

In particular, the simplest type of power function (B1) is used for the representation of the lightning channel base current in the IEC standard on Lightning Protection, IEC 62305, dealing with return stroke and subsequent strokes and s defined as follows:

$$i(t) = \frac{I_{max}}{\eta} \frac{\left(\frac{t}{T}\right)^{n}}{1 + \left(\frac{t}{T}\right)^{n}} e^{-t/\tau} \#(B2)$$

where I_{max} stands for the peak current, η is the peak current correction factor (to fit the experimental data), T is the rise time constant, τ is the fall time constant, and n is the steepness factor.

It is also useful to write (B2) as follows [18]:

$$i(t) = \frac{I_{max}}{\nu} x(t) y(t) \#(B3)$$

where:

$$x(t) = \frac{\left(\frac{t}{T}\right)^{n}}{1 + \left(\frac{t}{T}\right)^{n}} \#(B4)$$

is referred to as the *rise* equation and where:

$$y(t) = e^{-t/\tau} \#(B5)$$

is referred to as the *decay* function.

Some authors, e.g., in [19], define the correction factor as follows:

$$\eta = e^{-\frac{t}{T}\left(\frac{n\tau}{T}\right)^{1/n}} \#(B6)$$

Furthermore, in [20], [21], the summation of two Heidler exponential functions is implemented to represent the subsequent return stroke current as follows:

$$i(t) = \frac{I_{max,1}}{\eta_1} \frac{\left(\frac{t}{T_1}\right)^n}{1 + \left(\frac{t}{T_1}\right)^n} e^{-t/\tau_1} + \frac{I_{max,2}}{\eta_2} \frac{\left(\frac{t}{T_2}\right)^n}{1 + \left(\frac{t}{T_2}\right)^n} e^{-t/\tau_2} \#(B7)$$

Note that the expression given in (B7) could represent recurrent SARS-CoV-2 waves. Thus, it is appealing to state some definite mathematical similarities in modelling lightning discharge and spreading of SARS-CoV-2.

Here, one observes the active infected individuals over discrete time instead of current distribution over continuous time. The fitting procedure is performed by applying the least square method (LSM) to the assumed theoretical distribution given in (B2) and the total confirmed number of infections for the first spring COVID-19 wave to obtain unknown parameters which can hardly be biologically justified but achieves a satisfactory goodness-of-fit ($^2S = 23.1568$).

Furthermore, it would be necessary to correlate the lightning parameters in Heidler function expressions with standard COVID-19 parameters given in Appendix A. Entire correlation is not possible, as the Heidler function is not obtained as an exact solution. The Heidler function should be considered a practical mathematical expression to fit COVID-19 data successfully.

Nevertheless, analyzing some analytical solutions of epidemic models [22], [23], such as Susceptible-Infected-Recovered (SIR), some general correlations could be undertaken.

Thus, the cumulative number of cases C(t) versus time can be written as follows [23]

$$C(t) = I(t) + R(t) = I_0 \frac{\beta}{\beta - \gamma} e^{(\beta-\gamma)t} \#(B8)$$

where $I(t)$ is the number of infected individuals, while $R(t)$ stands for recovered or deceased individuals. In addition, β denotes the infection rate, while γ is related to the recovered/deceased individuals. Therefore, some correlations in the mathematical structure of (B2) and (B8) could be noticed.

Appendix C: Holt-Winters decomposition and predictive model

HWS model was based on a time series analysis module by Wessa, which was further adjusted for this study. The primary input parameters were the time series data (the number of positive cases), while additional modelling parameters included the expected seasonal pattern, level of smoothing and type of seasonal effects. The expected seasonal pattern was set at seven days to offset the weekly changes observed in the data. Smoothing was set at triple, meaning that all three components of the decomposed signal were smoothed. For purposes of this study, both additive and multiplicative models were compared; additive assumes a constant epidemic pattern, while multiplicative assumes an additional changing effect on top of the additive model.
